# Increased maternal consumption of methionine as its hydroxyl analog promoted neonatal intestinal growth without compromising maternal energy homeostasis

**DOI:** 10.1186/s40104-016-0103-y

**Published:** 2016-08-05

**Authors:** Heju Zhong, Hao Li, Guangmang Liu, Haifeng Wan, Yves Mercier, Xiaoling Zhang, Yan Lin, Lianqiang Che, Shengyu Xu, Li Tang, Gang Tian, Daiwen Chen, De Wu, Zhengfeng Fang

**Affiliations:** 1 Key Laboratory for Animal Disease Resistance Nutrition of the Ministry of Education, Animal Nutrition Institute, Sichuan Agricultural University, Ya’an, 625014 China; 2 Adisseo France S.A.S., CERN, Commentry, France

**Keywords:** Energy metabolism, Intestine, Lactating sows, Methionine nutrition, Neonatal pigs

## Abstract

**Background:**

To determine responses of neonatal intestine to maternal increased consumption of DL-methionine (DLM) or DL-2-hydroxy-4-methylthiobutanoic acid (HMTBA), eighteen primiparous sows (Landrace × Yorkshire) were allocated based on body weight and backfat thickness to the control, DLM and HMTBA groups (*n* = 6), with the nutritional treatments introduced from postpartum d0 to d14.

**Results:**

The DLM-fed sows showed negative energy balance manifested by lost bodyweight, lower plasma glucose, subdued tricarboxylic acid cycle, and increased plasma lipid metabolites levels. Both villus height and ratio of villus height to crypt depth averaged across the small intestine of piglets were higher in the DLM and HMTBA groups than in the control group. Piglet jejunal oxidized glutathione concentration and ratio of oxidized to reduced glutathione were lower in the HMTBA group than in the DLM and control groups. However, piglet jejunal aminopeptidase A, carnitine transporter 2 and IGF-II precursor mRNA abundances were higher in the DLM group than in the HMTBA and control groups.

**Conclusion:**

Increasing maternal consumption of methionine as DLM and HMTBA promoted neonatal intestinal growth by increasing morphological development or up-regulating expression of genes responsible for nutrient metabolism. And increasing maternal consumption of HMTBA promoted neonatal intestinal antioxidant capacity without compromising maternal energy homeostasis during early lactation.

## Background

The gut plays a key role not only in the digestion, absorption and metabolism of nutrients, but also in the immune surveillance of the intestinal epithelial layer and regulation of the mucosal response to foreign antigens [1]. There is growing evidence indicating that sulfur-containing amino acids (SAA), methionine and cysteine, play an important role in maintaining mucosal growth and antioxidant defense of neonatal intestine [2, 3]. The synthesis of the major cellular reductant, glutathione [4], and of the major extracellular reductant, cysteine [5], both depend on cysteine or its precursor, methionine [6]. SAA deficiency suppressing intestinal epithelial growth has been demonstrated in a previous study in neonatal pigs [2]. DL-methionine (DLM) and its hydroxyl analog, DL-2-hydroxy-4-methylthiobutanoic acid (HMTBA), are two methionine sources commonly used in commercial feed [7]. Our recent studies indicated that increased inclusion of methionine as HMTBA in diets of sows or piglets promoted milk synthesis [8], attenuated the detrimental effect of early weaning on piglet growth and ameliorated intestinal antioxidant capacity [9]. However, little is known about the association of pre-weaning intestinal growth status with maternal methionine nutrition.

The objectives of the present study were to determine whether neonatal intestinal growth could be promoted by increased maternal consumption of methionine as DLM or HMTBA. Sow plasma metabolites were analyzed by ^1^H nuclear magnetic resonance (NMR) spectroscopy method, and intestinal antioxidant capacity, morphology and expression of genes related to digestion, transport, metabolism, growth and immunity of piglets were also evaluated, which may provide a biological explanation for neonatal intestinal growth in response to maternal methionine nutrition.

## Methods

### Animals and diets

All procedures involving animals were approved by the Animal Care and Use Committee of Animal Nutrition Institute, Sichuan Agricultural University. Eighteen pregnant crossed (Landrace × Yorkshire) primiparous sows artificially inseminated with mixed semen from two Duroc boars were used in this experiment. On d 110 of gestation, sows were moved into farrowing crates (2.1 m × 0.7 m) with an area (2.1 m × 0.6 m) for newborn pigs on each side of the crate in an environmentally regulated farrowing house. Temperature in the farrowing house was maintained at 20 ± 1 °C, and heat lamps provided supplemental heat to the pigs. Sows were allocated based on body weight and backfat thickness to the control (CON), DLM and HMTBA groups (*n* = 6), with the nutritional treatments introduced from postpartum d0 to d14. The CON diet was formulated based on NRC (1998) [10] lactating-sow nutrition requirement (Table 1), and contained methionine and methionine + cystine at 0.25 % and 0.53 % of diet, respectively. The DLM and HMTBA diets were prepared by adding 0.134 % DLM and 0.151 % HMTBA on top of the CON diet, respectively, according to a previous study [8]. The time span of farrowing between the sows was within three days. Litter size was set within 12 h of farrowing for each sow to ten piglets by cross-fostering, and the body weight averaged across litters was 1.42 ± 0.02 (standard deviation) kg, 1.42 ± 0.03 kg and 1.44 ± 0.04 kg for the CON, DLM and HMTBA groups, respectively. Sows had free access to diets and water throughout the experimental period. Body weight of each sow was weighed before the morning meal at postpartum d0, d7 and d14. Feed intake of each sow was recorded daily. Body weight of suckling piglets were determined before the morning meal at postnatal d0 and d14.

**Table 1:** Ingredients and composition of the control diet of sows^a^

Ingredient	kg	Composition	Percent
Corn	582	Calculated protein and AA contents ^d^	
Wheat bran	60	CP	16.10
Soybean meal (CP 43 %)	240	Lysine	0.97
Fructose-glucose syrup	20	Methionine	0.25
Glucose	25	Methionine + Cystine	0.53
Soybean oil	35	Threonine	0.63
Lysine-HCl	1.45	Valine	0.83
Threonine	0.08	Analyzed AA contents	
Valine(99 %)	0.92	Total AA	16.39
Dicalcium phosphate	14.1	Lysine	1.01
Limestone	10.5	Methionine	0.30
Sodium chloride	4	Methionine + Cystine	0.61
Premix ^b, c^	5	Threonine	0.63
Choline chloride (50 %)	2	Valine	0.91
Total	1000.05		

^a^The DL-methionine (DLM) and DL-2-hydroxy-4-methylthiobutanoic acid (HMTBA) diets were prepared by adding 1.34 kg of DLM (99 %) and 1.51 kg of HMTBA (88 %), respectively, to 1000.05 kg of the control (CON) diet at the expense of corn. Each diet (per kg) contained 14,200 kJ digestible energy. CP: Crude protein. ^b^Provided per kg of diet: vitamin A, 12,000 IU; vitamin D_3_, 2800 IU; vitamin E, 100 mg; menadione, 3.5 mg; thiamine, 3.5 mg; riboflavin, 8.5 mg; niacin, 35 mg; d-panthothenic acid, 21 mg; vitamin B_6_, 3.5 mg; vitamin B_12_, 35 μg; d-biotin, 420 μg; folic acid, 2.5 mg. ^c^Provided per kg of diet: copper, 10 mg; iron, 120 mg; manganese, 30 mg; zinc, 80 mg; iodine, 0.21 mg; selenium, 0.23 mg; antioxidant, 100 mg; anti-mould additive 500 mg. ^d^Calculated according to NRC [10]

### Milk collection of sows

The milk samples (20 mL) from each sow were collected at postpartum d0 and d7 before the morning meal as described previously [11]. In brief, before manually milking functional pectoral and inguinal glands, piglets were separated from sows for 90 min firstly, then, each sow was injected with 10 I.U. oxytocin from the ear vein to collect milk samples which were stored at −20 °C until milk composition was analyzed.

### Tissue collection of offspring

At postnatal d14, one female suckling piglet approaching average body weight of the litter, from each sow was slaughtered by exsanguination as described [12]. After death, the abdomen was immediately opened and the entire intestine was rapidly removed, thoroughly flushed with ice-cold sterile saline to remove luminal chyme. Then, the intestine was dissected free of mesenteric attachments, and placed on a smooth and cold surface tray. Next, the middle site of duodenum, jejunum and ileum were obtained quickly as described [13]. Several two-cm-long sections of tissues were taken at a pre-determined distance from the jejunum, and frozen in liquid nitrogen for subsequent glutathione (GSH) and glutathione disulfide (GSSG) analysis and RNA isolation. Two-cm-long segments of duodenum, jejunum and ileum were sampled and immediately fixed in phosphate-buffered paraformaldehyde (4 %, pH 7.6) for histological measurements.

### Sow plasma metabolites by ^1^H NMR spectroscopy

Plasma samples were prepared by mixing 200 μL of plasma with 400 μL of saline containing 50 % D_2_O (for field frequence lock purposes). The proton NMR spectra of plasma were recorded at 298 K on a Bruker Avance DRX-600 spectrometer (Bruker Biospin, Rheinstetten, Germany) operating at a ^1^H frequency of 600.11 MHz with a triple-resonance, high-resolution probe. A water-presaturated Carr-Purcell-Meiboom-Gill (CPMG) pulse sequence (recycle delay–90 ^o^–(τ–180 ^o^–τ)_n_–acquisition) was used to attenuate NMR signals from macromolecules. The spin-spin relaxation delay (2n*τ*) of 200 ms was employed. Typically, 90 ^o^ pulse was set to 10.0 μs and 32 transients were collected into 32 k data points for each spectrum with a spectra width of 20 ppm. For assignment purposes, five two-dimensional (2D) NMR spectra including ^1^H-^1^H J-resolved spectroscopy (J-Res), ^1^H-^1^H correlation spectroscopy(COSY), ^1^H-^1^H total correlation spectroscopy(TOCSY), ^1^H-^13^C heteronuclear single quantum coherence spectroscopy (HSQC) and ^1^H-^13^C heteronuclear multiple bond correlation spectroscopy (HMBC) were acquired for selected samples.

The free induction decays were multiplied by an exponential window function with a 1 Hz line-broading factor prior to Fourier transformation. All NMR spectra were initially phase adjusted, and then the baseline was corrected by using Mestrenova 7.0 software (Mestrelab Research SL, Spain). Chemical shift was referenced to the peak of the methyl proton of L-lactate at δ 1.33.

NMR spectra (δ 0.5–9.5) were integrated into regions of 0.002 ppm wide by using Mestrenova 7.0 software (Mestrelab Research SL, Spain). Regions distorted by imperfect water saturation were discarded with the regions containing urea signals. These regions were δ 4.47–5.18, δ 5.5–6.0 and δ 4.28–4.45. Subsequently, each integral region was normalized to the total sum of all integral regions for each spectrum prior to pattern recognition analyses.

### Sow milk composition analysis

Frozen milk samples were thawed at 4 °C, and then 10 mL of each sample was used for milk fat, protein and solids-not-fat (SNF) analysis by a quick milk element analyzer (MILKYWAY-CP2, Hangzhou Simple Technology. Co., Ltd.).

### Antioxidant capacity of neonatal jejunal tissue

The jejunum samples were thawed at 4 °C and grinded on ice in glass homogenizer with 20 volumes (wt/v) of ice-cold physiological saline. After that, homogenates were centrifuged at 4,000 × *g* and 4 °C for 20 min, and then supernatants were collected for GSH and GSSG analysis. The GSH plus GSSG levels of the samples were determined by using a method as described [14]. Briefly, total glutathione was determined by following the rate of reduction of 5, 5′-dithiobis-2-nitrobenzoic acid (DTNB) by GSH at 412 nm and comparing this to a GSH standard curve. GSSG in the samples were detected by using the same method after treating samples with 4-vinylpyridine for 60 min. The GSH concentration was calculated using the total glutathione subtracted by twofold of GSSG.

### Intestinal morphology analysis of neonates

Histomorphometric analyses were performed on H&E-stained tissue sections as described [15]. In brief, the tissue samples were split along the mesentery and fixed smoothly then immersed in Davidson’s fixative (333 mL of 95 % ethanol, 220 mL of 37 % formaldehyde, 110 mL of glacial acetic acid, and 330 mL of distilled water) for 24 h. After that, the tissue samples were taken out from the fixative, cut into 1 cm^2^ sections, preserved in fresh fixative. Subsequently, they were cleared in xylene before being embedded in paraffin. Four cross-sections per tissue sample were stained with hematoxylin and eosin. Villus height and crypt depth were determined for 12 villi and crypts using the Nikon Eclipse 80I fluorescence microscope (Nikon company, Japan) equipped with an epi-fluorescence image analysis system. Villi and crypts were only measured when there was a complete longitudinal section of a villus and an associated crypt. The average villus height and crypt depth per slide was used as experimental observation [16].

### RNA extraction and real-time qPCR in neonatal intestinal tissue

As previously described in detail [17], a total of fifteen genes related to regulation of gene expression, signal transduction, aminosugar synthesis, protein and peptide degradation, lipid metabolism, intestinal transport, growth regulation and immune function were determined. The detected genes included *DNA-binding protein inhibitor ID-2*, *adenylate cyclase*, *N-Acyl-D-glucosamine 2-epimerase*, *aminopeptidase A*, *cathepsin F precursor*, *ubiquitin carboxyl-terminal hydrolase*, *acyl-CoA dehydrogenase*, *carnitine transporter-2*, *oxysterol binding protein-related protein 10*, *sodium- and chloride-dependent creatine*, *preprogalanin*, *somatostatin precursor*, *IGF-II precursor*, *vanin-1* and *leukocyte antigen related protein precursor*. Total RNA was extracted from the frozen samples by using the RNAiso Plus regent (TAKARA BIO INC., Shiga, Japan) according to the manufacturer’s specifications. Concentration of RNA in samples was quantified by using DU-800 nucleic and protein detector (Beckman, USA) at an OD of 260 nm, the ratio of OD_260_ to OD_280_ between 1.8 and 2.0 was acceptable. Integrity of the RNA was verified by electrophoresis on a 1 % agarose gel stained with ethidium bromide. Real time-qPCR analysis was performed by using the SYBR Green method and the ABI 7900HT Sequence Detection System. Briefly, first-strand cDNAs were synthesized from 1 μg of total RNA as described [12]. The thermal cycling parameters were as follows: 95 °C for 30 s, followed by 40 cycles at 95 °C for 15 s and 60 °C for 34 s, followed by 95 °C for 15 s, 60 °C for 1 min and 95 °C for 15 s. To confirm the specific amplification, melt curve analysis was performed and the Real time-qPCR products were also detected on ethidium bromide-stained 2 % agarose gel after electrophoresis using Tris-acetate-EDTA buffer. Primers for individual genes were designed using Primer Express 3.0 (Applied Biosystems, Foster City, CA, USA). Information about primer pairs for selected genes is summarized in Table 2. The standard curve of each gene was run in duplicate and three times for obtaining reliable amplification efficiency as we previously described [18]. The correlation coefficients (r) of all standard curves were > 0.99 and the amplification efficiency was between 90 % and 110 %. Relative mRNA abundances of the 15 determined genes in the jejunum samples were calculated by using the 2^-∆∆C^_T_ method [19] and all the data were normalized with the 18 SRNA [20] in the same samples.

**Table 2:** Accession number, primer sequence and product size of genes evaluated

Gene (accession number)	Primer sequence (5'–3')	Product size, bp
*18S ribosomal RNA* (NR_046261.1)		114
Forward	GACTCAACACGGGAAACCTCAC	
Reverse	ATCGCTCCACCAACTAAGAACG	
*DNA-binding protein inhibitor ID-2* (NM_001037965.1)		121
Forward	CCAGTGAGGTCCGTTAGGAAAA	
Reverse	GCTTGGAGTAGCAGTCGTTCAT	
*IGF-II precursor* (NM_213883.2)		163
Forward	CATCGTGGAAGAGTGCTGCT	
Reverse	CCAGGTGTCATAGCGGAAGAACT	
*Somatostatin precursor* (NM_001009583.1)		159
Forward	CTCTCCATCGTCCTGGCTCT	
Reverse	GTTCTCTGTCTGGTTGGGTTCAG	
*Vanin-1* (NM_214133.1)		142
Forward	GGATGCTTGAGAAGAGAGAAGACG	
Reverse	ACCGAGTCACCACAGGAGTGTA	
*Ubiquitin carboxyl-terminal hydrolase FAF-g *(NM_004654.3)		123
Forward	TGGTCTCCTCCAGTACAAAGCA	
Reverse	CTGGTCATCTGGCTCCTCTTCT	
*Adenylate cyclase* (NM_001114)		87
Forward	CACACTACTGCCCTTCAGCA	
Reverse	ATCAAAGAACCGAGCACCAG	
*N-Acyl-D-glucosamine 2-epimerase* (NM_213900.1)		91
Forward	CCTGCCTTACTGCGTCTCTT	
Reverse	TCAGGTAGCCAAACCATTCC	
*Carnitine transporter 2* (NM_003060)		120
Forward	GTTCCAAGAAGCAGCAGTCC	
Reverse	AAAGCCCAAAATAGCCCACT	
*Oxysterol binding protein-related protein 10* (NM_017784)		86
Forward	CCACCCATCTCCTGCTTCTA	
Reverse	CCCATGAACTTGCTTTTGGT	
*Acyl-CoA dehydrogenase* (NM_213897.1)		94
Forward	TTGGTGCCATAGCAATGACAGA	
Reverse	AACAGCCACTACAATCACAACATCA	
*Aminopeptidase A* (NM_214017.1)		73
Forward	TGGAGAACTGGGGACTCATCAC	
Reverse	TTAGCCACAAGTCTTCCCACCAT	
*Cathepsin F precursor* (TC104654)		75
Forward	AGAGCCGAGATAGCTTCCTGAAA	
Reverse	TCCTTCCCATTGGTTCTCACA	
*Leukocyte antigen related protein precursor* (NM_130440.2)		67
Forward	GCGAGGGATTCATTGACTTCAT	
Reverse	CTCAGAGTGATGAACACCCCG	
*Preprogalanin *(NM_214234 · 1)		94
Forward	CAACCACAGATCATTCCACGAC	
Reverse	GCAAGAAAGCCAGAAACTCCATT	
*Sodium-and chloride-dependent creatine transporter 1* (NM_001177327.1)		77
Forward	TCGACTACTACTCGGCCAGCG	
Reverse	ACCAGCGGGGTGAAGAAAGAC	

### Statistical analysis

Data are presented as means with pooled SEM, unless otherwise specified. The pen was considered the experimental unit for statistical analyses. Body weight, feed intake, milk composition, jejunal antioxidant and mRNA abundance data were analyzed by using the GLM procedures of SAS statistical package (version 8.1; SAS Institute, Inc.). The following statistical model was used: *Y*_*ij*_ = *μ* + *T*_*i*_ + *e*_*ij*_ where *Y* is the analyzed variable, *μ* is the overall mean, *T* is the effect of treatment (*i* = 1…3), and *e* is the residual error (*i* = 1…3, *j* = 1…6). The least significant difference test was used to compare the group means when the *F* test in the analysis of variance table was significant. Meanwhile, body weight at postpartum d7 was compared with that at postpartum d0 by a two-sample paired *t* test. Multivariate data analysis was carried out on the normalized NMR datasets with the software package SIMCA-P+ (v11 · 0, Umetrics, Sweden). Principal component analysis (PCA) was performed to show an overview of intrinsic similarity/dissimilarity within the data sets. The orthogonal projection to latent structure-discriminant analysis (OPLS-DA) was further carried out using the unit-variance scaled (UV) NMR data as X-matrix and class information as Y-matrix [21]. The quality of the model was assessed by the parameters R^2^X representing the total explained variations for X matrix and Q^2^ indicating the model predictability. The models were validated by two methods: a cross validation method and a permutation test [22, 23]. The models were interpreted by the coefficient coded loading plots. The loadings were backtransformed in Excel (Microsoft, USA) and plotted with color-coded absolute coefficient values (|r|) of the variables in MATLAB [22]. The coefficient plot indicated the significance of variables (resonances) that contributed to the differentiation of classes of interest. The significant discriminatory metabolites were indicated in red color whereas blue color showed no significance. In the present study, appropriate correlation coefficients were used as the cutoff values (depending on the number of animals used) for the statistical significance based on the discrimination significance at the level of *P* < 0.05; such was determined according to the test for the significance of the Pearson’s product–moment correlation coefficient [22]. *P* < 0.05 was considered statistical significance. As described by Littell et al. [24], the repeated-measures data for intestinal morphology was analyzed by using the MIXED procedures of SAS statistical package (version 8.1; SAS Institute, Inc.).

## Results

### Body weight and feed intake of sows, and body weight of piglets

**Fig. 1:**
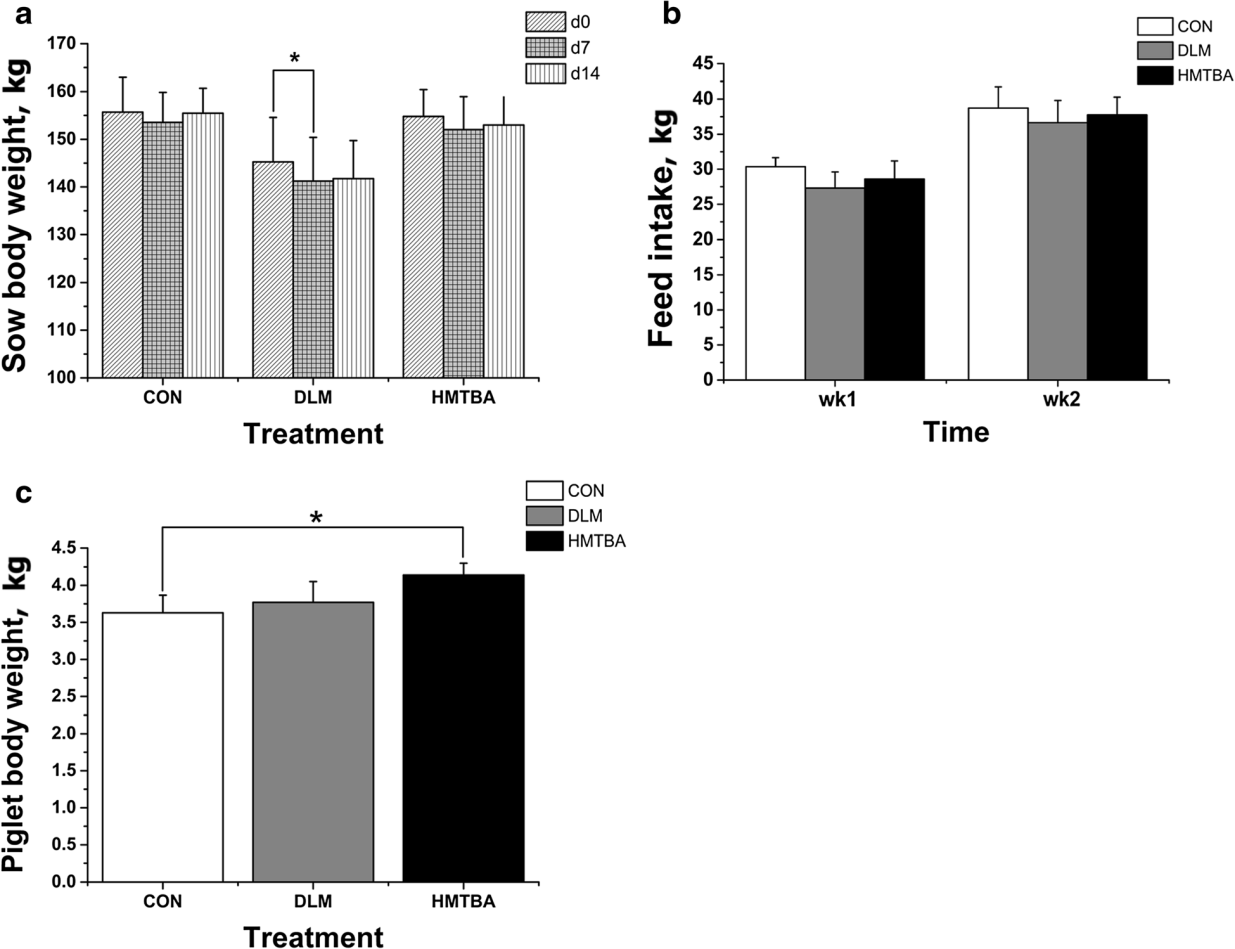
Body weight (**a**) and feed intake (**b**) of sows, and body weight of piglets (**c**). Partial data were edited from a published paper [8], to make sense that comparison of plasma metabolomics at postpartum d7 against at postpartum d0 and comparison of intestinal measurements among treatments at postnatal d14. Data were means ± SEM. Control (CON), DL-methionine (DLM), DL-2-hydroxy-4-methylthiobutanoic acid diets (HMTBA). ^*^Significant difference between day or treatments (*P* < 0.05)

Body weight of sows at postpartum d0, d7 and d14 were not different (*P* > 0.05) among treatment groups (Fig. 1a). Feed intake of sows during postpartum wk1 and wk2 was also not different (*P* > 0.05) among treatment groups (Fig. 1b). However, sows fed the DLM diet had decreased (*P* < 0.05) body weight at postpartum d7 compared with that at postpartum d0 (Fig. 1a). At postnatal d14, suckling piglets in the HMTBA group showed a higher (*P* < 0.05) bodyweight than those in the CON group (Fig. 1c).

### Milk composition

Milk composition including fat, protein and SNF was not different (*P* > 0.05) among treatment groups at postpartum d0 (Table 3). However, the HMTBA-fed sows had higher (*P* < 0.05) milk fat concentration than the DLM-fed sows at postpartum d7 (Table 3).

**Table 3:** Milk composition of lactating sows

	Treatment			
Item	CON	DLM	HMTBA	Pooled SEM
Postpartum d0				
Milk fat	6.07	6.28	5.76	1.02
Milk protein	7.79	7.75	7.55	1.14
Solid-not-fat	20.52	20.70	22.20	2.72
Postpartum d7				
Milk fat	7.23^ab^	6.66^b^	7.88^a^	0.57
Milk protein	3.89	3.79	3.99	0.12
Solid-not-fat	10.41	10.17	10.70	0.34

Control (CON), DL-methionine (DLM), DL-2-hydroxy-4-methylthiobutanoic acid diets (HMTBA). ^a, b^Within a row, means without a common superscript differ (*P* < 0.05)

### Multivariate data analysis result of NMR data

**Fig. 2:**
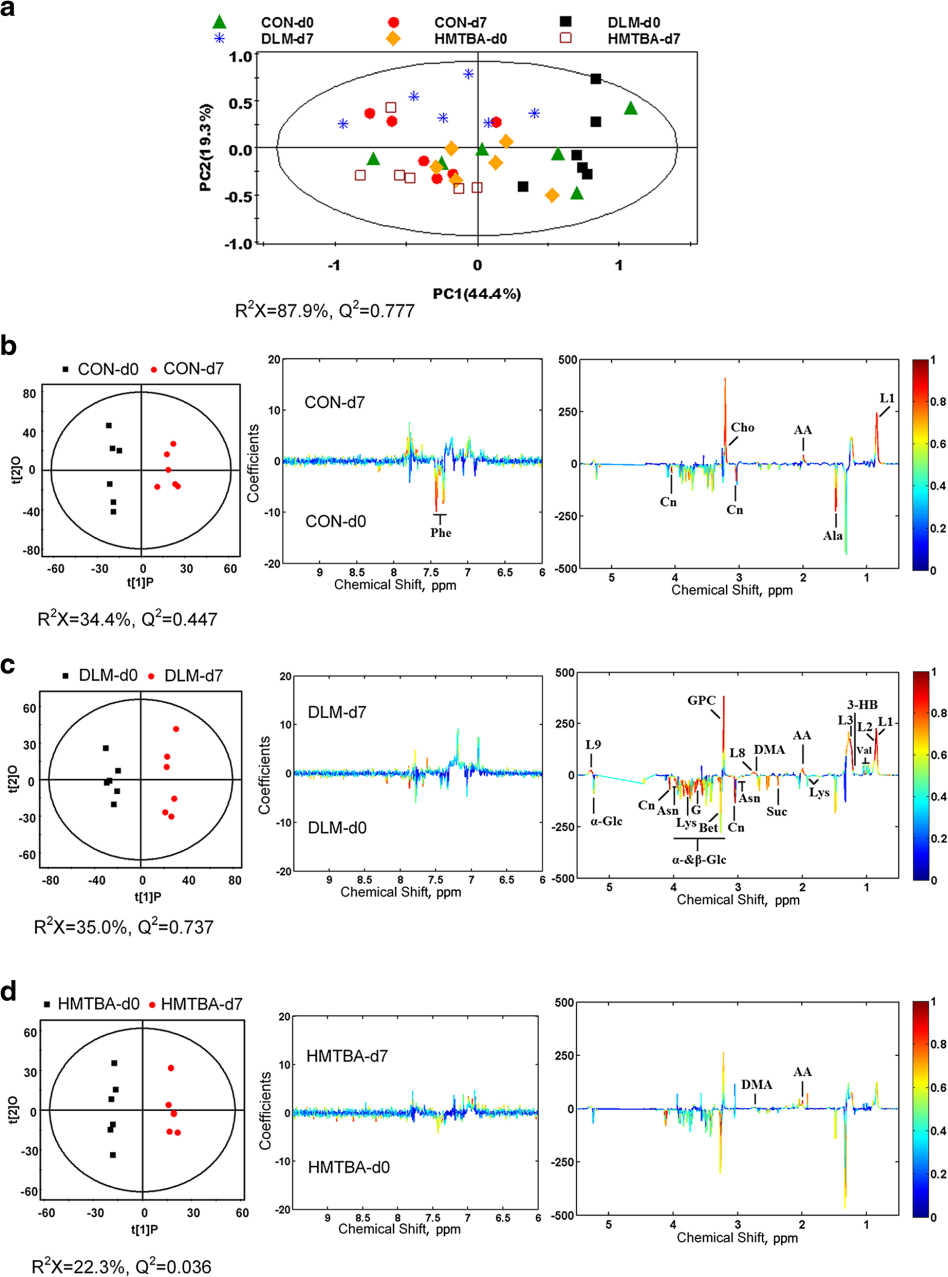
PCA (**a**) and OPLS-DA with the corresponding coefficient loading plots (**b**), (**c**), (**d**) score plots. The color map in the (**b**), (**c**) and (**d**) shows the significance of metabolite variations between the two classes. The peaks in the positive direction indicate the metabolites that are more abundant in the groups in the positive direction of the first principal component. The metabolites that are more abundant in the groups in the negative direction of the first primary component are presented as peaks in the negative direction. The significant discriminatory metabolites were indicated in red color whereas blue color shown no significance. Principal component analysis (PCA), orthogonal projection to latent structure-discriminant analysis (OPLS-DA), the plasma sample at postpartum d0 of sows fed the control diet (CON-d0), the plasma sample at postpartum d0 of sows fed DL-methionine diet (DLM-d0), the plasma sample at postpartum d0 of sows fed DL-2-hydroxy-4-methylthiobutanoic acid diet (HMTBA-d0), the plasma sample at postpartum d7 of sows fed the control diet (CON-d7), the plasma sample at postpartum d7 of sows fed DL-methionine diet (DLM-d7), the plasma sample at postpartum d7 of sows fed DL-2-hydroxy-4-methylthiobutanoic acid diet (HMTBA-d7), 3-hydroxybutyrate (3-HB), acetamide (AA), acetate (Ace), alanine (Ala), asparagine (Asn), betaine (Bet), choline (Cho), citrate (Cit), creatinine (Cn), creatine (Cr), 3, 4-dihydroxymandelate (DHM), dimethylamine (DMA), glycerol (G), glucose (Glc), glycerolphosphocholine (GPC). Low density lipoprotein, CH3-(CH2)n- (L1). Very low density lipoprotein, CH3-(CH2)n- (L2). Low density lipoprotein, CH3-(CH2)n- (L3). Lipid, =CH-CH2-CH = (L8). Lipid, −CH = CH- (L9). Lysine (Lys), myo-inositol (m-I), phenylalanine (Phe), succinate (Suc), valine (Val)

PCA was initially performed on the spectral data, and two principal components were calculated for the six treatment groups with a total of 44.4 % and 19.3 % of variables being explained by PC 1 and PC 2, respectively. The PCA results (Fig. 2a) showed that separations between postpartum d0 and d7 in sows were observed in their metabolic plasma profiles from the CON, DLM, or HMTBA groups, respectively. The plasma metabolic changes in the sows from postpartum d0 to d7 were analyzed by using the OPLS-DA strategy. A plot of OPLS-DA scores showed a clear separation between the sows at postpartum d0 and d7 (Fig. 2b, c, d). The corresponding coefficient plot showed that the plasma levels of acetamide, choline and low density lipoprotein (LDL) in the CON group were higher (*P* < 0.05) at postpartum d7 than at postpartum d0 (Fig. 2b and Table 4). By contrast, the plasma levels of alanine, creatinine, and phenylalanine in the CON group were lower (*P* < 0.05) at postpartum d7 than at postpartum d0 (Fig. 2b and Table 4). The corresponding coefficient plot showed that the plasma levels of 3-hydroxybutyrate, acetamide, dimethylamine, glycerophosphorylcholine, LDL, very low density lipoprotein (VLDL), lipids and valine in the DLM group were higher (*P* < 0.05) at postpartum d7 than at postpartum d0 (Fig. 2c and Table 4). However, the plasma levels of allantoin, asparagine, betaine, creatinine, glycerol, lysine, *myo*-inositol, succinate and glucose in the DLM group were lower (*P* < 0.05) at postpartum d7 than at postpartum d0 (Fig. 2c and Table 4). The corresponding coefficient plot showed that the plasma levels of acetamide and dimethylamine in the HMTBA group were higher (*P* < 0.05) at postpartum d7 than at postpartum d0 (Fig. 2d and Table 4).

**Table 4:** OPLS-DA coefficients derived from the NMR data of metabolites in plasma obtained from different treatments

Metabolite	Correlation coefficient^a^		
	CON-d7 (vs CON-d0)	DLM-d7 (vs DLM-d0)	HMTBA-d7 (vs HMTBA-d0)
3-Hydroxybutyrate	-	0.884	-
Acetamide	0.856	0.796	0.824
Alanine	−0.792	-	-
Allantoin	-	−0.757	-
Asparagine	-	−0.899	-
Betaine	-	−0.831	-
Choline	0.816	-	-
Creatinine	−0.902	−0.913	-
Dimethylamine	-	0.787	0.843
Glycerol	-	−0.842	-
Glycerophosphocholine	-	0.851	-
Lipid, CH_3_-(CH_2_)_n_-(LDL)	0.892	0.915	-
Lipid, CH_3_-(CH_2_)_n_-(VLDL)	-	0.859	-
Lipid, CH_3_-(CH_2_)_n_-(LDL)	-	0.837	-
Lipid, =CH-CH_2_-CH=	-	0.825	-
Lipid, −CH = CH-	-	0.886	-
Lysine	-	−0.822	-
*myo*-Inositol	-	−0.874	-
Phenylalanine	−0.780	-	-
Succinate	-	−0.785	-
Valine	-	0.894	-
α-Glucose	-	−0.888	-
β-Glucose	-	−0.853	-

^a^Correlation coefficients (r), positive and negative signs indicate positive and negative correlation in the concentrations, respectively. The correlation coefficient of│r│ > 0.755 was used as the cutoff value for the statistical significance based on the discrimination significance at the level of *P* = 0.05 and *df* (degree of freedom) = 5. “-” means the correlation coefficient│r│ is less than 0.755. The plasma sample at postpartum d0 of sows fed the control diet (CON-d0), the plasma sample at postpartum d7 of sows fed the DL-methionine diet (CON-d7), the plasma sample at postpartum d0 of sows fed the DL-methionine diet (DLM-d0), the plasma sample at postpartum d7 of sows fed the DL-methionine diet (DLM-d7), the plasma sample at postpartum d0 of sows fed the DL-2-hydroxy-4-methylthiobutanoic acid diet (HMTBA-d0), the plasma sample at postpartum d7 of sows fed DL-2-hydroxy-4-methylthiobutanoic acid diet (HMTBA-d7), low density lipoprotein (LDL), very low density lipoprotein (VLDL)

### Antioxidant index of suckling piglets

At postnatal d14, jejunal GSH, GSSG and the ratio of glutathione disulfide to glutathione (GSSG/GSH) of suckling piglets were not different (*P* > 0.05) between the DLM and CON groups (Table 5). However, piglets in the HMTBA group had lower (*P* < 0.05) jejunal GSSG concentration and GSSG/GSH than those in the CON and DLM groups at postnatal d14 (Table 5).

**Table 5:** Jejunal glutathione and glutathione disulfide concentration at postnatal d14 of suckling piglets

Item	Treatment			Pooled SEM
	CON	DLM	HMTBA	
GSH (μmol/g protein)	41.01	39.70	41.37	2.68
GSSG (μmol/g protein)	3.09^a^	2.93^a^	1.40^b^	0.24
GSSG/GSH	0.072^a^	0.074^a^	0.036^b^	0.01

Control (CON), DL-methionine (DLM), DL-2-hydroxy-4-methylthiobutanoic acid diets (HMTBA), glutathione (GSH), glutathione disulfide (GSSG), the ratio of glutathione disulfide to glutathione(GSSG/GSH). ^a, b^Within a row, means without a common superscript differ (*P* < 0.05)

### Intestinal morphology of suckling piglets

**Fig. 3:**
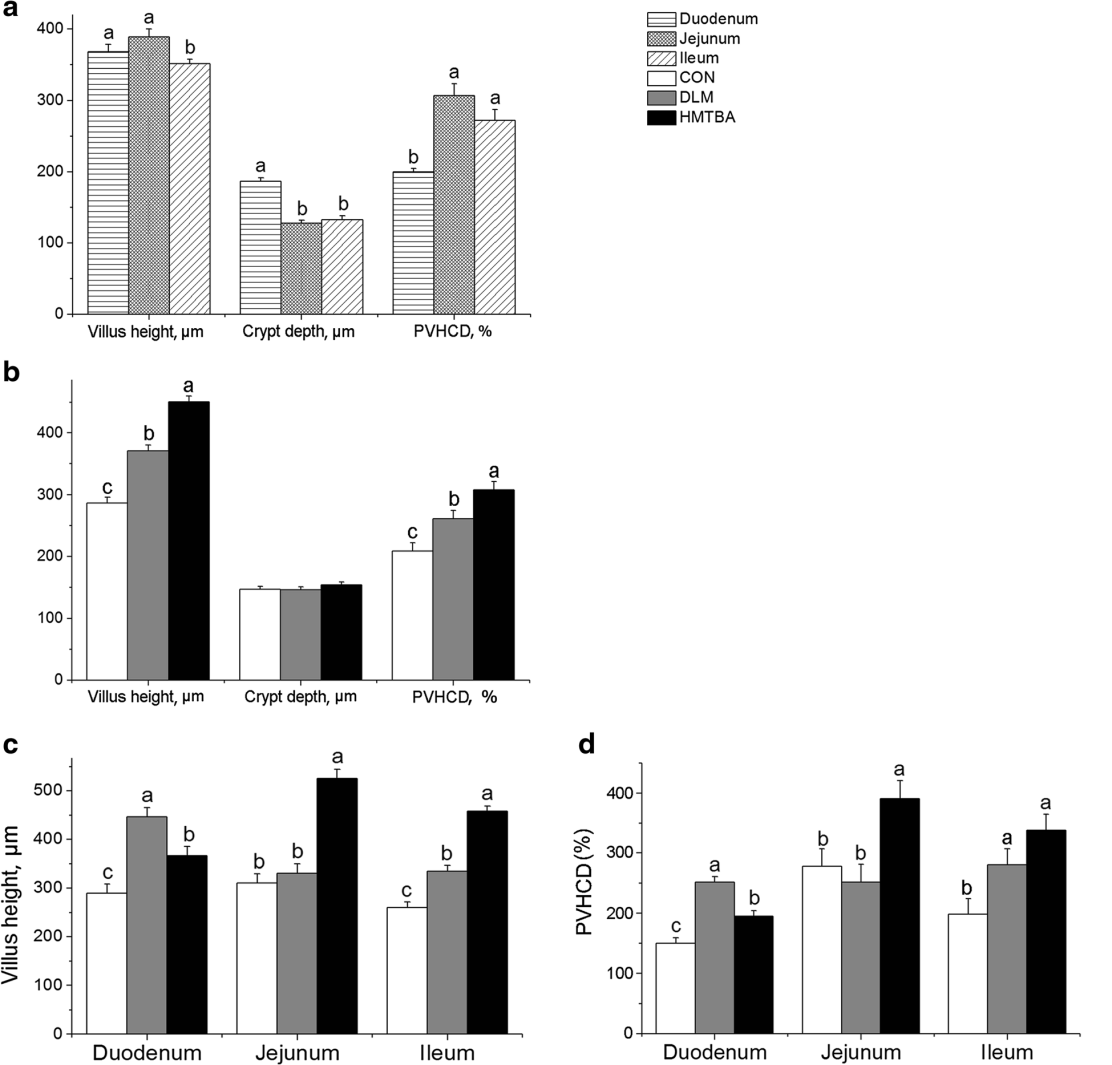
Effects of maternal methionine nutrition on intestinal morphology of 14 days old suckling piglets. Effects of intestine site on intestinal morphology (**a**), effects of dietary treatment on intestinal morphology (**b**) and effects of intestine site × dietary treatment interaction on intestinal morphology (**c**), (**d**). Data were means ± SEM. Control (CON), DL-methionine (DLM), DL-2-hydroxy-4-methylthiobutanoic acid diets (HMTBA), percentage of villus height to crypt depth (PVHCD). ^a, b, c^Means for same genes with no common letters differ (*P* < 0.05)

At postnatal d14, dietary treatment, intestinal site and diet × site interaction all had effect (*P* < 0.05) on intestinal morphology (Fig. 3). Both villus height and percentage of villus height to crypt depth (PVHCD) averaged across the small intestine of piglets were higher (*P* < 0.05) in the DLM and HMTBA than in the CON groups (Fig. 3b). Specifically, increased maternal consumption of methionine as DLM enhanced (*P* < 0.05) duodenal and ileal villus height (Fig. 3c) and PVHCD (Fig. 3d) of suckling piglets. In contrast, villus height (Fig. 3c) and PVHCD (Fig. 3d) in the duodenum, jejunum and ileum of suckling piglets were all higher (*P* < 0.05) in the HMTBA group than in the CON group. The HMTBA group also had higher (*P* < 0.05) jejunal and ileal villus height than the DLM group, while duodenal villus height is lower (*P* < 0.05) in the HMTBA than in the DLM group (Fig. 3c).

### Genes expression in suckling piglet jejunum

**Fig. 4:**
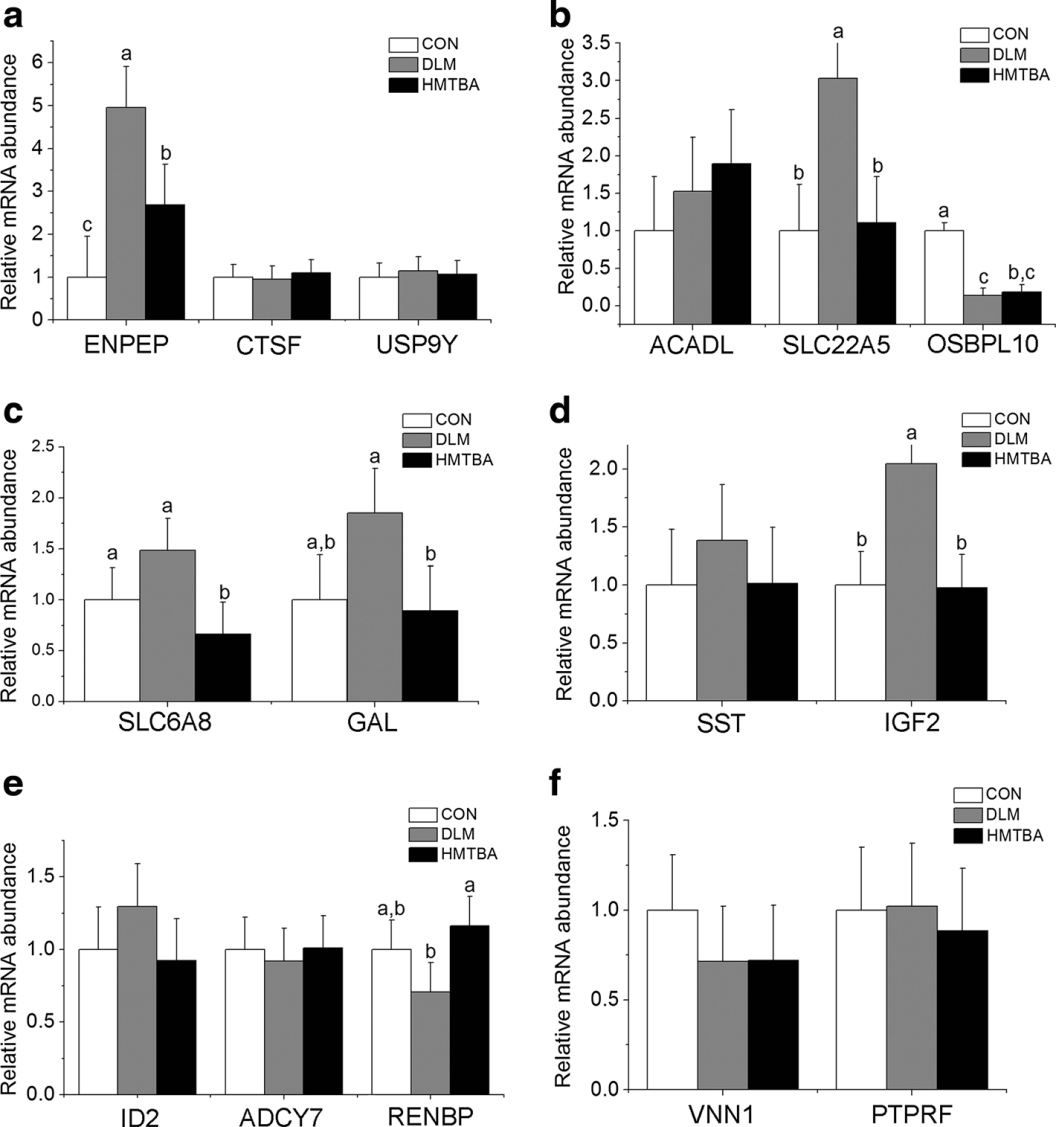
Effects of maternal methionine nutrition on gene expression in jejunum of 14 days old suckling piglets. The relative mRNA abundance of genes related to protein and peptide degradation (**a**), the relative mRNA abundance of genes related to lipid metabolism (**b**), the relative mRNA abundance of genes related to intestinal transport genes (**c**), the relative mRNA abundance of genes related to growth regulation (**d**), the relative mRNA abundance of genes related to the regulation of gene expression, signal transduction and aminosugar synthesis (**e**) and the relative mRNA abundance of genes related to immune function (**f**). Data were means ± pooled SEM. Control (CON), DL-methionine (DLM), DL-2-hydroxy-4-methylthiobutanoic acid diets (HMTBA), aminopeptidase A (ENPEP), cathepsin F precursor (CTSF), ubiquitin carboxyl-terminal hydrolase FAF-γ (USP9Y), acyl-CoA dehydrogenase (ACADL), carnitine transporter 2 (SLC22A5), oxysterol binding protein-related protein 10 (OSBPL10), sodium- and chloride-dependent creatine transporter 1 (SLC6A8), preprogalanin (GAL), somatostatin precursor (SST), IGF-II precursor (IGF2), DNA-binding protein inhibitor ID-2 (ID2), adenylate cyclase (ADCY7), N-acyl-D-glucosamine 2-epimerase (RENBP), vanin-1 (VNN1), leukocyte antigen related protein precursor (PTPRF). ^a, b, c^Means for same genes with no common letters differ (*P* < 0.05)

The mRNA abundances of aminopeptidase A (Fig. 4a), carnitine transporter 2 (Fig. 4b), preprogalanin (Fig. 4c), and IGF-II precursor (Fig. 4d) were higher (*P* < 0.05) in the DLM group than in CON and HMTBA groups. Sodium- and chloride-dependent creatine transporter 1 mRNA abundance (Fig. 4c) was also higher (*P* < 0.05) in the DLM group than in the HMTBA group. The HMTBA group also had higher (*P* < 0.05) N-acyl-D-glucosamine 2-epimerase mRNA abundance (Fig. 4e) than the DLM group. Oxysterol binding protein-related protein 10 mRNA abundance (Fig. 4b) was higher (*P* < 0.05) in the CON group than in DLM and HMTBA groups.

## Discussion

The first endpoint of this study was to determine how the sows respond to increased consumption of methionine as DLM or HMTBA during early lactation. Notably, milk fat content was found to be lower in the DLM group than in the HMTBA group at postpartum d7. However, it appeared that there was no difference either in body weight at postpartum d7 or in feed intake during postpartum wk1 among the three treatment groups. Considering that the coefficient of variation of bodyweight among sows within a treatment group was up to 16 %, the potential difference of bodyweight change might have been masked due to the relatively small replicate numbers. Therefore, a paired *t* test was further used to compare the body weight at postpartum d7 against at postpartum d0 in each treatment group. Intriguingly, only DLM-fed sows showed lost body weight, which might account for the difference in milk composition among treatment groups. Given that physiological metabolism is influenced in lactating sows using body reserve to satisfy lactation [8], the metabolomics of plasma at postpartum d7 against at postpartum d0 was further analyzed, which may provide further biochemical evidence for the change of body reserve. An important finding in the present study was that the DLM-fed sows had reduced plasma levels of betaine and creatinine, but increased plasma level of dimethylamine at postpartum d7 compared with those at postpartum d0. It has been indicated that creatine is involved in energy metabolism in vertebrates. The conversion of creatine to creatinine is a spontaneous and nonenzymatic process [25]. Betaine is a methyl donor to facilitate methionine synthesis from homocysteine [26], thereby improving biosynthesis of creatine [27]. However, methionine is also methyl donors of methylamine to form dimethylamine [28]. Another significant finding was that there were decreased plasma levels of glucose, glycerol, *myo*-inositol and succinate but increased plasma levels of 3-hydroxybutyrate, lipoprotein and lipids in the DLM-fed sows at postpartum d7 compared with those at postpartum d0. It is well known that glucose can be completely catabolized as energy source via the tricarboxylic acid cycle, but it also generates glycerol to synthesize lipid [29]. A previous study indicated that *myo*-inositol deficiency resulted in increased fatty acid mobilization from adipose tissue [30]. Meanwhile, when triglycerides are presented as lipoproteins, it can be cleaved by endothelial lipoprotein lipase and converted into ketone body such as 3-hydroxybutyrate in liver [31]. Skeletal muscle or other tissues will give priority to utilize ketone body as an energy source when glucose consumption is reduced [32]. We can infer that the energy release from the tricarboxylic acid cycle is depressed by reduced concentrations of glucose and succinate. It was therefore proposed that the increased plasma levels of 3-hydroxybutyrate, lipoproteins and lipids and reduced glycerol and *myo*-inositol contents were a result of reduced level of glucose available for energy supply. Taken together, the compromised creatinine production and tricarboxylic acid cycle along with increased lipid catabolism and 3-hydroxybutyrate production illustrated that the DLM-fed sows might be in negative energy balance during the first wk postpartum. This notion was further supported by body weight loss of the DLM-fed sows at the first wk postpartum. In contrast, compared with postpartum d0, there was no change in plasma energy related metabolites and body weight of the HMTBA-fed sows at postpartum d7, suggesting that energy metabolism of lactating sows during early lactation was not affected by increased consumption of methionine as HMTBA.

Milk is the major nutrients source for suckling piglets, and the intestine plays a key role in the digestion, absorption and metabolism of nutrients [1]. The HMTBA-fed sows appeared to have higher milk fat content at postpartum d7 than the DLM-fed sows and higher milk fat and lactose content at postpartum d14 than the DLM- and CON-fed sows [8]. Accordingly, higher body weight of suckling piglets was observed in the HMTBA group at postnatal d14 [8]. Thus the second endpoint of this study was to determine whether neonatal intestinal growth could be affected by increased maternal consumption of methionine as DLM or HMTBA. A previous study has indicated that carnitine deficiency was associated with carnitine transporter 2 deficiency, which give rise to a mitochondrial fatty acid oxidation problem thereby inhibiting lipid and energy metabolism [33, 34]. Piglets in the DLM group had up-regulated intestinal expression of carnitine transporter 2, suggesting enhanced energy expenditure in the intestine. In support of this view, there was extended expression of sodium- and chloride-dependent creatine transporter 1 which mediates intestinal uptake of creatine [35]. Piglets in the DLM group also had higher expression of IGF-II precursor which regulates the expression of the IGF-II [36], and IGF-II is supposed to trigger intestinal growth [37]. However, piglets in the DLM group did not show higher jejunal villus than those in the CON or HMTBA groups. It was therefore proposed that the up-regulated expression of genes responsible for energy metabolism might be a result of intestinal compensatory growth.

Glutathione is a momentous intracellular peptide with antioxidant defense, and the ratio of GSSG/GSH is shown to be a good measure of oxidative stress of an organism. It was observed in our study that piglets reared by the HMTBA-fed sows had lower jejunal GSSG content than those reared by the CON-fed sows and, moreover, piglets in the HMTBA group had the lowest GSSG/GSH, indicating improved antioxidant capacity of neonatal intestine by increased maternal consumption of methionine as HMTBA. Previous studies have shown that oxidative stress accelerates degeneration of the intestinal epithelium [38], but a reduced redox potential maintains the proliferative state of intestinal epithelium [39]. Thus, we proposed that the increased jejunal villus height and the ratio of villus height to crypt depth in piglets reared by the HMTBA-fed sows were associated with the increased jejunal antioxidant capacity. In contrast, jejunal GSSG content and GSSG/GSH in piglets reared by the DLM-fed sows remained to be the same as that in the CON group. It appeared that increased maternal consumption of methionine as HMTBA could improve intestinal antioxidant capacity of suckling piglets. This might be associated with higher milk fat which could be used as an energy source to satisfy intestinal growth thereby benefiting intestinal health.

Aminopeptidase A, an important digestive enzyme [40], can split off protein and peptide [41]. Noting that aminopeptidase A mRNA abundance was lower in the CON than in the DLM and HMTBA groups, we proposed that increased maternal consumption of methionine might promote degradation and subsequent absorption of milk protein by neonatal pigs. Oxysterol binding protein-related protein 10 has been implicated in sterol containing cholesterol transport [42, 43], and low level of cholesterol is considered good for small intestinal microvillus membrane fluidity [44]. In this regard, decreased expression of oxysterol binding protein-related protein 10 following increased maternal consumption of methionine as DLM and HMTBA might suggest the facilitation of nutrients transport in the small intestine. However, given that galanin derived from preprogalanin inhibits intestinal smooth muscle activity directly [45, 46], increased maternal consumption of methionine as DLM might compromise neonatal intestinal motility due to the up-regulated expression of preprogalanin. N-acyl-D-glucosamine 2-epimerase is involved in bioconversions of N-acetyl-D-glucosamine to N-Acetyl-D-neuraminic acid [47], the derivatives of which play a prominent role in antiviral agents [48]. The up-regulated expression of N-acyl-D-glucosamine 2-epinerase suggested that the intestine of piglets in the HMTBA group might have greater intestinal disease resistance capability than those in the CON and DLM groups.

## Conclusions

The DLM-fed sows were in negative energy balance during the first week postpartum. Meanwhile, though increasing maternal consumption of methionine as DLM and HMTBA promoted neonatal intestinal growth by increasing morphological development or up-regulating expression of genes responsible for nutrient metabolism, suckling piglets in DLM group emerged intestinal compensatory growth and underpowered intestine motility, probably. In addition, increasing maternal consumption of methionine as HMTBA promoted neonatal intestinal antioxidant ability.

## Abbreviations

2D, two-dimensional; CON, control; COSY, correlation spectroscopy; CPMG, Carr-Purcell-Meiboom-Gill; DLM, DL-methionine; DTNB, 5, 5′-dithiobis-2-nitrobenzoic acid; GSH, glutathione; GSSG/GSH, the ratio of glutathione disulfide to glutathione; GSSG, glutathione disulfide; HMBC, heteronuclear multiple bond correlation spectroscopy; HMTBA, DL-2-hydroxy-4-methylthiobutanoic acid; HSQC, heteronuclear single quantum coherence spectroscopy; J-Res, J-resolved spectroscopy; LDL, low density lipoprotein; NMR, ^1^H nuclear magnetic resonance; NSF, solids-not-fat; OPLS-DA, orthogonal projection to latent structure-discriminant analysis; PCA, principal component analysis; PVHCD, percentage of villus height to crypt depth; SAA, sulfur-containing amino acids; TOCSY, total correlation spectroscopy; UV, unit-variance scaled; VLDL, very low density lipoprotein
